# Got Mylk? The Emerging Role of Australian Plant-Based Milk Alternatives as A Cow’s Milk Substitute

**DOI:** 10.3390/nu12051254

**Published:** 2020-04-28

**Authors:** Yianna Y. Zhang, Jaimee Hughes, Sara Grafenauer

**Affiliations:** 1 School of Agriculture and Food, Faculty of Veterinary and Agricultural Sciences, The University of Melbourne, Parkville, VIC 3052, Australia; 2 CSIRO Agriculture & Food, 671 Sneydes Road, Werribee, VIC 3030, Australia; 3 Grains & Legumes Nutrition Council, Mount Street, North Sydney, NSW 2060, Australia; 4 School of Medicine, University of Wollongong, Northfields Avenue, Wollongong, NSW 2522, Australia

**Keywords:** plant-based alternatives, milk, milk alternatives, nutrient composition, vegetarian, fortification

## Abstract

Growing ethical, environmental and health concerns have encouraged demand for novel plant-based milk alternatives, yet it remains nebulous whether these products are nutritionally adequate as cow’s milk replacements. The aim of this study was to conduct a cross-sectional survey of plant-based milk alternatives available in major Australian supermarkets and selected niche food retailers from November 2019 to January 2020 and assess two dietary scenarios (adolescents and older women) where dairy serves were substituted for plant-based alternatives against Australian Estimated Average Requirements (EAR). We collected compositional data from nutrition panels in juxtaposition with derivatives from the Australian Food Composition database, with a total of 115 products, including tree nuts and seeds (*n* = 48), legumes (*n* = 27), coconut (*n* = 10), grains (*n* = 19) and mixed sources (*n* = 10). Just over 50% of products were fortified, but only 1/3 contained similar calcium content to cow’s milk. Indiscriminate substitutions might reduce intakes of protein and micronutrients, particularly vitamin A, B2, B12, iodine and zinc, and lead to reductions >50% of the EARs for protein, zinc and calcium in the chosen dietary scenarios. To avoid unintended dietary outcomes, it is vital that consumers make pragmatic decisions regarding dietary replacements for cow’s milk.

## 1. Introduction

The consumption of cow’s milk can be traced back to the 7th century BC, with a definitive role in food production and cultures worldwide [1,2]. Against a backdrop of ethical, environmental and health concerns, there has been a surge of consumer interest in milk alternative beverages (also termed ‘mylk’), derived from aqueous extracts of plant ingredients, including nuts, legumes and grains [3]. Australia is witnessing significant growth in the plant-based milk alternatives market, with products accounting for ~7% of all milk consumed and estimated value of $230 million [4]. By virtue of their positive perception and high sensory acceptance [5,6], plant-based milk alternatives broaden avenues for serving the needs of diverse populations that harbour concerns associated with dairy. Its consumption coheres with the growing prevalence of health conditions, including milk protein allergy and lactose intolerance [7,8], consumer demand for vegan/vegetarian foods [9,10] and concerns with animal welfare [11]. Moreover, the resource and waste-intensive nature of industrial dairy production are indisputable [12,13], leading many consumers to perceive plant-based milk alternatives as being expedient over cow’s milk in environmental sustainability [14,15]. There have been multiple comparative life cycle assessments (LCAs) suggesting substantially fewer greenhouse gas emissions, eutrophication, water and land use during production [16,17,18], although some plant-based milks can have a higher ecological impact based on per kg of protein produced (e.g. almond [17]). 

Besides environmental quandaries, nutritional adequacy is also an important dimension of sustainable diets worldwide [19,20]. Cow’s milk is an economical yet nutrient-dense source of energy, containing highly bioavailable proteins with essential vitamins and minerals, as well as bioactive compounds, such as peptides and lipids [21]. An irrefutable diet staple, the average Australian reportedly consumes approximately 100 litres annually, constituting 62% of the total dairy intake [22,23]. The latest representative Australian nutrition survey (National Nutrition and Physical Activity Survey, 2011−12) demonstrated that milk ingestion constituted for ~20% of total calcium, 8.7% of phosphorous, 17.3% of iodine, 5.2% of vitamin A, 4.6% of B6 and 17.5% of B12 intake in the general population [24], a finding also replicated in a Canadian cohort [25]. Observational and dietary modelling studies have shown cow’s milk consumption to be a reliable indicator of dietary quality across multiple age groups, with consumers more likely to meet their requirements of protein, essential minerals and vitamins A, B2, B6 and B12 [26,27,28,29,30,31]. An ideal alternative would provide comparable levels of these nutrients, without the need to elicit major changes in dietary patterns to meet requirements. However, there appears to be a general consumer mistrust of the food supply [32] and public misconceptions over plant-based beverages and their nutrition credentials as ‘healthier’ alternatives [4].

Cow’s milk is commonly featured in dietary guidelines as a multifaceted contributor to nutrients, promoting bone health (e.g. in the USA [33] and Canada [25]). It offers a valuable supply of highly metabolisable calcium, protein and zinc, which are synergistic contributors to bone tissue and collagen synthesis [34,35]. The physiological requirements for these nutrients are at a pinnacle during critical stages of life. This includes children and adolescents, where 40% of an individual’s total bone mass begins to progressively accrue until peak mass is reached later in life [36]. Another is the expanding aging population, where diminishing bone density becomes common in individuals reaching 40, leading to elevated risks of osteoporosis and subsequent fractures [37,38]. Menopausal women, in particular, experience hormone-related changes that accelerate bone loss, such as in estrogen and Follicle Stimulating Hormone [39,40]. Eschewing cow’s milk for alternative plant-based sources are thus particularly relevant for these age groups to help ensure sufficient nutrient intakes. However, current Food Standards Australia and New Zealand (FSANZ) legislations only require a mandatory advisory warning on plant-based beverages as a milk substitution for children aged 5 and under [41].

Despite possessing functional properties in their own right, the composition inventories in the scientific literature highlight the inadequacies of unfortified plant-based milk alternatives in comparison to cow’s milk [42,43,44,45]. Milk alternatives occupy compositional flexibility, the formulation of which are governed under legislations distinct to cow’s milk [46,47]. Food legislations possess a country-level dependence, indicating that data generalisation across geographical locations may not be prudent, with differences in the ingredients and fortificants permitted. Little has been reported in relation to the state of Australian products, despite FSANZ making further approvals for voluntary fortification in plant-based milks in recent years [48]. The market availability of food products is a compelling driver underpinning consumer access and consumption [49,50]. Beyond a direct beverage, cow’s milk is an important culinary constituent, with 14% of its total ingestion in Australia originating from café style coffee [24]. The juxtaposition of these factors necessitates an evaluation of products available from supermarkets, cafés and health food stores. 

The aim of this study was to conduct a cross-sectional survey of plant-based milk alternatives available in Australian major supermarkets and selected niche food retailers (café’s and health food stores) from November 2019 to January 2020, collecting compositional data from nutrition panels in comparison to cow’s milk. This study helped to identify potential nutritional ramifications of replacing cow’s milk with plant-based milk alternatives through dietary modelling in adolescents (aged 12–18 years of age) and older women (aged 51+ years), in line with the Nutrient Reference Values [51]. Strategies to mitigate the nutritional gaps identified during our analyses were discussed with reference to some health-promoting constituents of cow’s milk. 

## 2. Materials and Methods 

A cross-sectional survey of plant-based milk alternatives was conducted between November 2019 and January 2020 in Metropolitan areas of Melbourne and Sydney. The data collection process replicated a previously recognised procedure [10,52]; however, the examination included three market categories: retail supermarkets, health food stores and local independent cafes with non-dairy milk offerings. The following major supermarkets were surveyed: Woolworths Group, Coles, Aldi and Independent Grocers of Australia (IGA), which collectively constitute 80.1% of the Australian grocery market share [53]. For each supermarket, a minimum of three stores within 30 km of each city’s Central Business District (CBD) were visited. A total of six independent health food stores, three from each city, and ten local café retailers, five from each city were also visited within the CBD. With permission from store managers, smartphones were used to collect on-pack nutrition information, including nutrition information panels (NIP) and ingredient lists. Plant-based milk alternatives from both refrigerated and long-life sections were included in the audit, where their intended use and quantity were reasonably presumed to be similar to cow’s milk (i.e. the product is displayed in juxtaposition to cow’s milk and can be directly consumed as a beverage). Products excluded from the auditing process included tinned coconut milks that were clearly intended for culinary use, and coconut water, a beverage composed of coconut liquid endosperm. Flavoured plant-based milk alternative products were also excluded, as their dairy-based counterparts are considered discretionary [54]. Data collected from all stores were screened to avoid product duplication. Collected nutritional information was validated using online data provided by the retailer or manufacturer’s website. 

To capture nutrition composition data for individual plant-based milk alternative products, three sources of nutrition information were referenced to. Where available, values were directly obtained from the NIP during the auditing process, as provided by the manufacturer of each product. Where insufficient data was available from the NIP, such as for some micronutrients in non-fortified products, values were factorially derived based on a percentage of the characterising ingredient, using composition data from the FSANZ Food Composition Database [55]. The latter option was made feasible by legal requirements of FSANZ Act 1991 that the characterising ingredient must be declared on products as a percentage of the final food product [56]. This percentage was then used to compute nutritional values per 100 grams. For example, a product containing 3% almonds, without additional fortificants, was considered to be nutritionally equivalent to 3 grams of unprocessed almonds per 100 mL of beverage. A list of the ingredient accession numbers used for calculations are provided in Appendix A. For vitamin A, retinol equivalents were used for standard comparison between sources, as milk is a source of both preformed vitamin A and carotenoids [57]. Nutrient values for dairy products were obtained from the Food Composition Database as controls (See Appendix A for accession numbers).

Data entries were transcribed from photographs, collated into a Microsoft Excel spreadsheet (Redmond, WA, USA) as Pivot tables and categorised by botanical source (Table 1). The data transfer process was cross-validated by a separate researcher. For each group, the total solids content was approximated by adding the macro- and micro-nutritional constituents per 100 mL, after unit adjustments. The products were also categorised based on whether they were sweetened or fortified (Table 2).

### 2.1. Modelling of Dietary Scenarios 

The nutritional ramifications of replacing present national recommendations of dairy intake with plant-based alternatives, in milk equivalents (1 serve being equal to 250 mL), were examined for two population groups—adolescents aged 12 to 18 and older women above the age of 50. These age groups have special physiological demands that are duly served by dairy food ingestion, including changes in dietary energy, and for nutrients important for bone health (protein, calcium, zinc). Nutrients derived from milks were computed as a fraction of the individual’s daily dietary requirements, in accordance with the Nutrient Reference Values (NRVs) in Australia. The NRV provides Estimated Energy Requirements (EER) and Estimated Average Requirement (EAR) for protein, calcium and zinc, which differs amongst age groups, gender and physical activity [51].

The adolescent scenario was based on modelling of the 3.5 serves of dairy foods recommended for both males and females aged 12–18, with the corresponding EAR computed for both genders at moderate physical activity level (PAL, 1.8). The older women scenario was projected based on 4 dairy food serves advised for females aged 51, with an average height of 1.6 meters [59], and light PAL (1.6) in agreement with previous reports for Australians of older age, including women [60,61]. The medians for each plant-based or cow’s milk group was utilised to compute values as a fraction of the EAR. 

### 2.2. Statistical Analysis

The nutrients subjected to statistical analyses included energy, macronutrients (protein, sugars, total and saturated fats, dietary fibre) and micronutrients (vitamin A, riboflavin, cobalamin, zinc, sodium and iodine). All statistical analyses were performed using MATLAB (Version 9.7.0.1190202, R2019b, The MathWorks Inc., Natick, MA, USA). Normality within each nutrient category was examined using the Kolmogorov–Smirnov method. Comparisons amongst categories were applied using the non-parametric Kruskal–Wallis test due to uneven sample size, with α < 0.05. The Dunn–Sidak approach was used for multiple comparisons against the control group (dairy/cow’s milk).

## 3. Results

**Figure 1:**
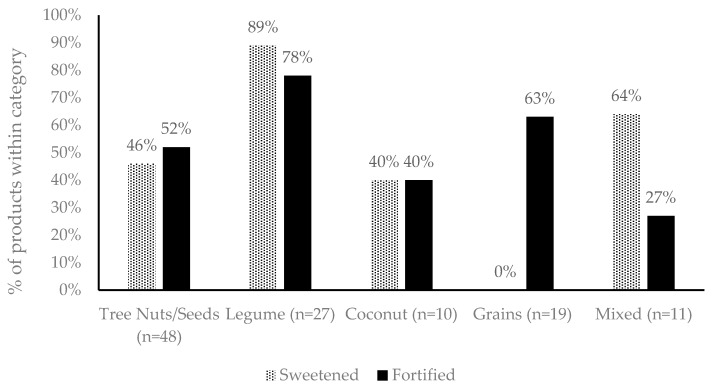
The proportion of products within each category that added sweeteners or fortificants in accordance with the ingredients list.

Following exclusion of product duplicates, a total of 115 independent products were examined, including 48 milks derived from tree nuts and seeds, 27 legume milks, 19 grain milks, 10 coconut milks and 11 milks with mixed ingredients (including almond and coconut and almond and cashew milks). The percentage of characterising ingredient in plant-based milk alternatives ranged from 2%–11% in tree nuts and seeds, 3.5%–16% in legumes, 7.5%–20% in coconut, 7%−16% in grains and 3.5%–15% from mixed sources. Recommended serving sizes for plant-milk products, in accordance with the nutritional panel, ranged from 200 to 250 mL, similar to the 250 mL found for cow’s milk. The approximate solids content ranged from 2.14 to 12.87 per 100 mL, compared to 9.46 to 13.34 in dairy food equivalents. Product ingredient lists revealed that, overall, 50% of the plant-based milk alternatives were sweetened, and 57% were fortified with micronutrients (Figure 1), but only 1/3 contained similar calcium content to cow’s milk. 

### 3.1. Nutrient Composition

**Figure 2:**
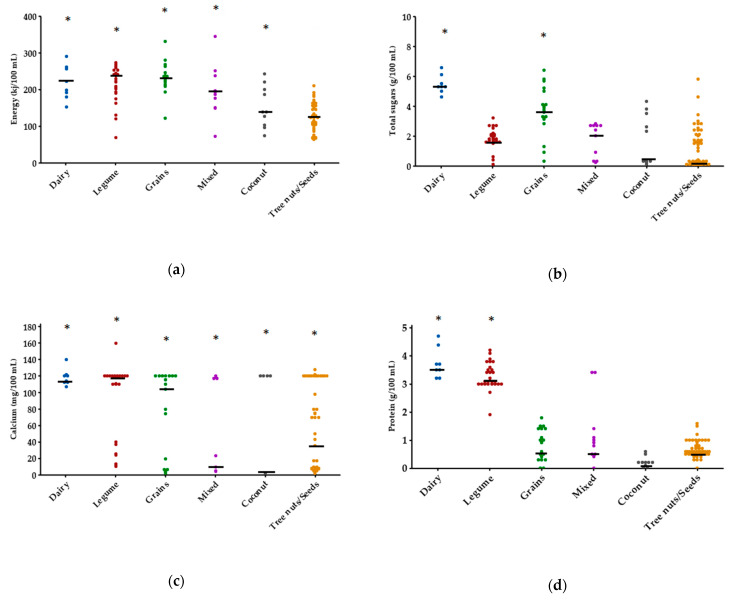
Energy and nutrient contents of Australian plant-based milk alternative products across categories: (**a**) Energy; (**b**) Total sugars; (**c**) Calcium; (**d**) Protein. Median values are outlined in black. Data were analysed using Kruskal–Wallis and Dunn–Sidak’s multiple comparison tests; groups not labelled with an asterisk (*) are significantly different (*p* < 0.01) from the control mean (dairy/cow’s milk).

The key constituents within milk, the distribution of energy, protein, total sugars and calcium content per 100 mL are illustrated in Figure 2. The overall energy content of plant-based milk alternatives ranged from 63–346 kJ/100 mL contrasted to 152–290 kJ/100 mL in cow’s milk. Tree nuts was the only group to demonstrate significantly (*p* < 0.01) lower energy, with a median of 123 versus 210 kJ/100 mL in cow’s milk. The protein content of milk alternatives ranged from 0 to 4.2 g/100 mL versus 3.2 to 4.7 g/100 mL from cow’s milk, with median values of 0.9 g (grains), 0.9 g (mixed), 0.2 g (coconut), 0.6 g (tree nuts/seeds) and 3.1 g (legume) per 100 mL. All plant-based groups apart from legumes were significantly (*p* < 0.01) inferior protein sources compared to cow’s milk (median: 3.6 g/100 mL). While the grains group exhibited no significant difference (*p* > 0.05) with cow’s milk in total sugars (median: 3.7 versus 5.3 g/100 mL), the legume, mixed, coconut and tree nuts/seed groups were comparatively lower in sugars, with median values ranging from 1.1–2.4 g/100 mL. 

No significant differences were found in the calcium concentrations of various plant milks when compared to cow’s milk counterparts (Figure 2). This coincided with a high prevalence of calcium fortification amongst all milk alternatives (42.6% of products examined) in the form of mineral salts, including calcium carbonate, phosphates, chloride and hydroxide, as per the ingredients list on the packaging. 

Table 3 presents the nutritional composition of other key macro- and micronutrients present in cow’s milk, including dietary fats (total and saturated), fibre and minerals (sodium, zinc, phosphorous and iodine) and vitamins (retinol equivalents, riboflavin and cobalamin), as median and range per 100 mL. The analyses demonstrated that milks from tree nuts/seeds had significantly less saturated fat compared to cow’s milk (median: 0.2 versus 0.85 g/ 100 mL, *p* < 0.01). Phosphorous levels in milks from tree nuts/seeds, coconut and grains were significantly less than that of cow’s milk (medians: 3–30 versus 94.5 mg/100 mL). No significant differences were found in the fat, dietary fibre or sodium content of plant-based milk alternatives as compared to cow’s milk. 

Despite the fortification of some products, plant-based milk alternatives were found to be poor sources of some micronutrients. Milks derived from tree nuts/seeds, coconut and grains showed significantly lower levels of retinol equivalents compared to cow’s milk (median: 0 versus 18.5 µg/100 mL). All groups contained little to no vitamin B12, except for mixed sources (median: 0.4 µg/100 mL). In relation to zinc, tree nuts/seeds and coconut milks were substandard sources versus cow’s milk (medians: 0.02–0.109 versus 0.39 mg/100 mL). Grain milks were the only source with iodine levels statistically comparable to cow’s milk, although a reduction was still observed (median 5.2 versus 24.85 µg/100 mL). None of the products examined was fortified with zinc or iodine, although one legume-based product contained kelp, a rich source of iodine. 

#### Comparisons with Cow’s Milk Under Australian Legislations

In accordance with FSANZ regulations, plant-based beverages that contain lower levels of protein than milk (≤3%) are unsuitable as a complete replacement for children under five years of age, while beverages with adequate protein but comparatively lower fat than cow’s milk (≤3.2%) are deemed inappropriate for children under the age of two [56]. Of the plant-based alternatives examined, only 27 (23.48% of total) contained the minimum protein concentration, 25 of which originated from legumes. Only 14 (12.17%) products contained fat concentrations equivalent to cow’s milk, and nine products (7.8%) met the criteria for both protein and fat concentrations. 

### 3.2. Projected Dietary Scenarios

Table 4 and Table 5 present the changes in energy, protein, sugars, calcium and zinc intake associated with substituting cow’s milk with plant-based sources in the context of the overall diet, assuming the recommended intakes for cow’s milk are met. Our modelling suggested that cow’s milk was a significant source of protein, calcium and zinc in adolescents and older women, providing 50% or over of the daily recommended intake in both population groups. 

In adolescents, the replacement of plant-based milk alternatives from legume, grains and mixed sources provided similar energy compared to cow’s milk (range: 12%−19% versus 13%−17% of EER), while tree nuts/seeds and coconut milks delivered less (range: 8%−11% of EER). Cow’s milk consumption provided 64%−90% of the protein EAR in this age group, which was similar to that from legumes (range: 55%−78%) but considerably higher than other plant sources (4%−23%). The same trend was observed for calcium, where legumes were the only comparable source to cow’s milk (98% EAR for both). Legume, grain and mixed milks provided slightly lesser zinc compared to cow’s milk (range: 15−36 versus 31%−58%), while milks derived from tree nuts/seeds and coconut were inadequate sources (2%−16% EAR). Regardless of the source, the sugars associated with milk ingestion constituted less than 5% of total energy in adolescents. This was well within the World Health Organization (WHO) guidelines of <10% of total energy coming from added sugars [62].

In older women, legume, grain and mixed milks provided similar quantities of energy to cow’s milk (range: 24−29 versus 27% of EER), while milks from tree nuts/seeds and coconut were lesser (16%−18%). Findings for protein and zinc were aligned with those from adolescents, with legumes being the only comparable protein source to cow’s milk (84% versus 97% EAR), and plant sources providing lesser quantities of zinc (3%−38% versus 61% EAR). Plant-based milk alternatives offered reduced sugars as a fraction of the total energy required for this age group (2−8 versus 11% in cow’s milk). Older women were likely to meet their calcium intake by selecting legume or grain-based drinks, which provided over 100% of the EAR, similar to in cow’s milk. 

## 4. Discussion

Bovine milk is known to be a nutrient-dense food with vast cultural acceptance, which is accompanied by a highly bioavailable delivery matrix. Although numerous plant-based replacement products are available, our study demonstrated considerable variation between sources, particularly in relation to protein, vitamin A, riboflavin, cobalamin, iodine and zinc. These findings reiterated those previously reported regarding the composition of plant-based milk alternatives from Spain [63], USA [44], Canada [43], Switzerland [64] and India [65], where legume-based (soy) were the only beverages that provided comparable values of protein to cow’s milk (approximately 3%). However, some demonstrated disparity in fortification patterns, supporting our hypothesis of possible geographic dependency on the composition of plant-derived milks. For example, a cohort of Spanish and Italian plant milks showed complete absence of fortification [63]. Fortification status is a significant determinant of micronutrient composition, as many plant-derived ingredients are endogenously low in nutrients, such as iodine and cobalamin [66]. 

Despite several successful nutrition interventions through mandatory fortification (e.g. with folate [67]), micronutrient deficiencies remain a contemporary public health issue in Australia [68,69], with prevalence up to 25% in some areas [70]. As a staple food, the substitution of cow’s milk with non-fortified plant-based alternatives may thus pose micronutrient displacement in populations with suboptimal dietary intake. These high-risk subgroups include those with restricted income and geographical access to food, such as in indigenous Australians [71,72] and rural/remote residents [73]. For example, inadequate zinc intake has been reported in these high-risk groups [74], which can lead to impairments in immunity and cognitive development even at mild levels [75]. Similarly, although the iodine status of Australians has generally improved following the mandatory use of iodised salt in flour for bread making [76], disparities in intake still exist between populations [72,73]. Iodine is imperative in thyroid hormone production and neurodevelopment, and its deficiency is a risk factor for mental impairment and birth defects [77]. In the current analysis, we were unable to identify products fortified with iodine apart from one product, indicating that there is only endogenous iodine present from the characterising ingredient. Low endogenous iodine levels in non-fortified products were poignantly highlighted in a study of products available in the UK [78]. However, the Australian case is likely associated with a previous public health issue in 2008, where numerous consumers had reported thyroid dysfunction associated with soy milk enriched with seaweed as a source of iodine [79].

Dairy food consumption during childhood and adolescence has been linked to increased bone density in adulthood [80], while effectively helping to decrease bone loss in pre- and post-menopausal women [40]. Results of our dietary modelling highlighted that by substituting cow’s milk with some plant-based beverages in these age groups, the intake of some bone-health promoting nutrients, including protein, calcium and zinc, could be reduced. This is a disconcerting discovery, as consumers reportedly perceive plant-based milk alternatives to be mineral-rich and good for bones [5,6,81]. The benefits derived from cow’s milk are likely attributable to the complex interactions between nutrients that synergistically promote bone and muscle anabolism, rather than solely as a source of essential minerals in bone formation itself [82], for example, calcium and Vitamin D. During stages of growth, the accrual of bone density is closely correlated with muscle mass [83], and muscle contraction is involved in maintaining bone density throughout life. Inadequate consumption of protein, calcium, zinc and possibly other nutrients, when substituting plant-derived products, maybe concerning in relation to both muscle and bone mass accrual in vulnerable age groups. Given that Australians currently obtain 7.1% of their protein intake from dairy foods [84], substitution with plant-derived proteins, even at the same quantity, may impact anabolic effects compared to those derived from animal sources [85,86]; however, these dietary components may be made up from other foods provided within the whole diet. Furthermore, plant-based milk alternatives may be limited in certain amino acids compared to bovine milk, which has a high biological value. In older women, where musculoskeletal aging is characterised by progressive, elevated obligatory losses in muscle and bone density [39,87], reduced calcium absorption efficiency may be more problematic [88]. Insufficient dietary intake to meet increased physiological demands can thus pose significant risks to the development of osteosarcopenia (co-occurrence of osteoporosis and sarcopenia), a chronic geriatric syndrome associated with increased mortality and morbidity [89,90], particularly as overall energy needs are lower at this stage of life. As specific health-promoting features of cow’s milk begin to unravel, they can be applied to plant milk matrices in the design of more nutritionally comparable substitutes for these consumers. Examples include the prospective role of cholecalciferol (vitamin D), medium-chain fatty acids and branched-chain amino acids in muscle anabolism [91,92,93]. 

Plant-based milk alternatives have long been designed to meet the needs of specialised populations, such as consumers avoiding lactose, saturated fats and cholesterol [94,95]. However, it is critical that consumers with pre-existing health conditions are made aware of the limitations associated with substitution. For example, nut-based beverages have minimal protein but are a source of phosphorous due to the natural occurrence in addition to as a food additive. The presence of highly bioavailable phosphate additives, as well as the high phosphorous-to-protein ratio, may not be ideal in the diets of individuals with chronic renal disease [96,97], a condition affecting 1.7 million Australians [98]. While grain-based milks, such as from rice, have been demonstrated to have a high glycaemic index due to enzymatic hydrolysis during production [99], making these products potentially unsuitable for diabetics. In previous studies, the lower energy and fat content relative to cow’s milk have been found to be triggers for plant milk consumption [6,81]. Indeed, we found some beverages to have low approximate solids content, which likely translated to lower energy, but potentially at the cost of reduced satiety relative to cow’s milk. This could lead to unwarranted energy compensation with discretionary foods [100], a finding previously observed in Australian young adults with poor calcium intake [101]. 

Fortification appears to be a promising strategy to mitigate the limitations in the micronutrient composition of plant-based milks, which we have in this range of products. However, food matrix interactions should be systematically studied to augment the concentrations of these nutrients, notably to levels present in cow’s milk. In cow’s milk, many micronutrients are present in a delivery matrix that is bioavailable during human digestion [21,102], in contrast to plant-derived matrices [103]. For example, the plant-based milk alternatives in our study predominantly contained comparable concentrations of calcium to cow’s milk, but the bioavailability of this calcium is not well understood. Minerals, such as calcium, possess a maximum concentration in which it stays soluble in a liquid medium, which is important for intestinal absorption as the excess tends to form non-bioaccessible precipitates. However, calcium concentrations in cow’s milk can exceed its maximum solubility due to their sequestration in casein phosphopeptides, a micellar protein absent in non-dairy products [104]. The high concentrations of calcium from milk can thus remain soluble during digestion for uptake, being progressively released for absorption as the casein slowly hydrolyses [105]. Plant-based milks, which provide varying food matrices, may not provide this advantage. Nascent investigations in vitro have displayed broad disparity in the levels of bioaccessible calcium found in fortified plant-based milks from rice, cashew, almond, peanut, coconut, oat and soy, likely due to the various types of fortificants used and the presence of inhibitory compounds [106,107,108]. Early isotope human studies demonstrated that soy milk fortified with calcium carbonate led to similar absorption levels from cow’s milk, although this was lower when tricalcium phosphate was used [109]. This demonstrates that bioavailability is likely dependent on both the food matrix and the type of fortificants used. Although no other human studies have been conducted on plant-based milk matrices, to the best of our knowledge, a recent rodent study showed that supplementation with bovine or soy milk led to positive effects on bone health, while consumption of an almond beverage led to detrimental effects despite containing the equivalence of calcium [110]. These preliminary findings highlight the need for further evaluations of different plant matrices that, in combination with suitable fortificants, provide efficient, bioavailable delivery of key nutrients. 

A series of approaches can be exploited by food technologists to compensate for some nutritional limitations identified in our study. This is particularly vital for milks, such as coconut, in our study, which was the least nutritionally adequate plant-based milk alternative, containing significantly less zinc, phosphorous, vitamin A, B2, B12 and iodine compared with cow’s milk. In addition to fortification, microbial synthesis of plant-based milks can effectively acquire selected B-group vitamins and, principally, in the chemically unbound form where high bioavailability is anticipated [111,112]. As examples, fermentation of soy by *Rhizopus* spp. and wheat by *Lactococcus* spp. has been shown to produce a minimum two-fold increase in riboflavin [113,114]; while many strains of genus *Lactobacilli* have been identified as cobalamin producers [115]. To address low levels of lipophilic nutrients, such as vitamin A, encapsulation using lipid-based nano-emulsions has been demonstrated to be bioavailable delivery systems [116,117]. Such an approach allows retinols and carotenoids to be solubilised in the same manner as they occur in the dairy lipid fraction [57]. Proteins are generally lower in plant-based milks, with crude nuts, seeds, grains and legumes, and possess limiting amino acid profiles, as well as restricted digestibility [118,119]. This can be ameliorated by complementing plant protein varieties to achieve a balanced amino acid profile [120], attempts of which we observed in the ‘mixed’ category of our study. 

Our cross-sectional evaluation was the first to be exclusively conducted on plant-based milk alternatives in Australia, a growing segment of the market with rising positive perception over their putative health and ecological benefits. Although we attempted to capture the litany of products available to Australian consumers, including those found in cafes, it would be worthwhile monitoring this product segment for new products or line extensions as it might have implications for the way such products are named and regulated. This study was limited by the use of predominantly manufacturer-supplied information and modelling, the accuracy of which could be validated in future by independent laboratory analyses. It is also likely that values of phosphorous were underestimated by modelling due to its frequent presence as a food additive (e.g. as dipotassium phosphate, pyrophosphates and phosphoric acid).

## 5. Conclusions

This cross-sectional survey of plant-based milk alternatives found substantial variability in the nutritional content of plant-based milk alternatives compared with cow’s milk, supporting previous works from other geographical locations. Based on the nutrients we examined, legume-based (pea and soy) milks were the most nutritionally sound when compared to other varieties. In the absence of mandatory fortification, prudent consumer selection of appropriately formulated products, regardless of the ingredient categories, is vital to avoid potential issues with reduced nutrient intake associated with substitution of cow’s milk. This includes not only nutritional inadequacies commensurate to lower intake and/or bioavailability but also the casual consequences linked to displacement of dietary energy. Due to the high proportion of consumers selecting plant-based milk alternatives for non-dietary reasons, it is recommended that current legislations implement further advisory labelling, particularly for older women and adolescents. Additional formulations should target these age groups, taking into account protein content and quality, among adequate quantities of micronutrients, such as zinc and calcium. Nonetheless, in an age of plant substitution over animal products, prospective gaps in the nutritional value of plant-based milk alternatives must not be overlooked, particularly where bioavailability remains equivocal.

## Figures and Tables

**Table 1:** Classifications of plant-based milk alternatives in the current study.

Group	Ingredients Included
Tree nuts and seeds	Almond, Cashew, Macadamia, Hazelnut, Hemp
Legume	Soybean, pea
Coconut	Coconut Milk/Cream
Grains	Rice, Oat, Quinoa
Mixed ^1^	Almond, Hazelnut, Coconut Cream, Oat, Cashew, Faba, Pea, Rice, Chia

^1^ A combination of two or more of the ingredients listed.

**Table 2:** Definitions used within the current study.

Term	Definition
Sugars	Total sugars present in the product per 100 mL. Includes both free and intrinsic hexose monosaccharides and disaccharides. ^1^
Sweetened	A product, containing one of the following ingredients: cane sugar, brown rice syrup, agave or tapioca syrup. ^2^
Fortified	A product, containing micronutrients in addition to those intrinsically present in the original material, which are permitted for voluntary addition into plant-based beverages by FSANZ. Includes vitamin A, riboflavin (B2), cobalamin (B12), vitamin D, calcium, phosphorous and iodine. Added protein isolates were excluded from this definition.

^1^ Reference: FSANZ [58], ^2^ Grain milks, whose high carbohydrate and sugar content is usually subject to enzymatic hydrolysis during production, were excluded from this definition.

**Table 3:** Selected macro- and micronutrient values of plant-based milk alternatives per 100 mL.

	Cow’s Milk		Tree Nuts/Seeds		Legumes		Coconut		Grains		Mixed Sources	
	*Median*	*Range*	*Median*	*Range*	*Median*	*Range*	*Median*	*Range*	*Median*	*Range*	*Median*	*Range*
Total fat (g)	1.4	0–3.5	1.9	0.8–3.1	2.8	0–3.5	2.2	1.8–3.7	1.2	0–3.4	2.9	1.5–6.6
Saturated fat (g)	0.85	0–2.3	0.2 *	0–0.4	0.3	0–0.8	2.1	1.4–3.4	0.17	0–0.5	0.5	0–4.8
Dietary fibre (g)	0	0–0.1	0	0–1.9	0	0–1.4	0	0–0.9	0	0–1.3	0	0–1
Zinc (mg)	0.39	0.12–0.48	0.11 *	0.03–0.69	0.19	0.14–0.66	0.02 *	0.02–0.06	0.22	0.07–0.37	0.25	0.09–0.26
Sodium (mg)	40.5	36–47	44	22–140	57	16–93	44.5	20–100	58	37–110	46	9–110
Phosphorous (mg)	94.5	85–119	17.05 *	5–115.5	87.75	31.04–108	3 *	2–8	30 *	16–87	73	26.1–103
Retinol equivalents (µg)	18.5	0–54	0 *	0–110	0.64	0–82	0 *	0	0 *	0	0 *	0
Riboflavin/Vitamin B2 (µg)	0.21	0.16–0.23	0.0024 *	0–0.17	0.04	0.004–0.42	0 *	0	0.001 *	0–0.002	0.007	0.003–0.17
Cobalamin/Vitamin B12 (µg)	0.6	0.5–0.8	0 *	0–0.4	0 *	0–0.9	0 *	0	0 *	0	0.4	0–0.4
Iodine (µg)	24.9	18.7–29.1	0 *	0	0 *	0–1.4	0.15 *	0.1–0.4	5.2	0–11.8	0 *	0–5.6

An asterisk (*) indicates statistically different values from the cow’s milk control (*p* < 0.01), as analysed by Kruskal–Wallis and Dunn–Sidak’s multiple comparison tests.

**Table 4:** Projected dietary scenarios of cow’s and plant-based milk consumption, based on current Australian guidelines of 3.5 dairy food serves for adolescents (both male and female, aged 12−18). One serving is equivalent to 250 mL.

	Energy (kJ)	% EER ^1^	Protein (g)	% EAR	Sugars (g)	% Energy	Calcium (mg)	% EAR	Zinc (mg)	% EAR
Cow’s milk	1838	13–17	32	64–90	46	0.3–7	1024	98	3.5	31–58
Tree nuts/Seeds	1076	8–10	5.3	11–15	9.6	0.1–2	407	39	1	9–16
Legumes	2004	14–18	27	55–78	16	0.1–2	1033	98	1.7	15–28
Grains	2021	14–19	7.9	16–23	32	0.2–5	910	87	1.9	18–32
Coconut	1216	9–11	1.8	4–5	11	0.1–2	5.3	0.5	0.2	2–3
Mixed	1689	12–15	7.9	16–23	21	0.2–3	82	8	2	20–36

^1^ EER = Estimated Energy Requirements, EAR = Estimated Average Requirements.

**Table 5:** Projected dietary scenarios of cow’s and plant-based milk consumption, based on current Australian guidelines of 4 dairy food serves for older women (aged 51 and above). One serving is equivalent to 250 mL.

	Energy (kJ)	% EER ^1^	Protein (g)	% EAR	Sugars (g)	% Energy	Calcium (mg)	% EAR	Zinc (mg)	% EAR
Cow’s milk	2100	27	36	97	53	11	1170	106	3.95	61
Tree nuts/Seeds	1230	16	6	16	11	2	465	42	1.09	17
Legumes	2290	29	31	84	18	4	1180	107	1.9	29
Grains	2310	29	9	24	37	8	1040	95	2.2	34
Coconut	1390	18	2	5	13	3	6	0.6	0.2	3
Mixed	1930	24	9	24	24	5	94	9	2.5	38

^1^ EER = Estimated Energy Requirements, EAR = Estimated Average Requirements.

## Appendix A

**Table A1:** List of ingredients’ accession numbers used for modelling from the Australian Food Composition Database.

Ingredient	Number
F006081	Nut, almond, with skin, raw, unsalted
F006088	Nut, cashew, raw, unsalted
F006099	Nut, macadamia, raw, unsalted
F006098	Nut, hazelnut, raw, unsalted
F004002	Flour, soya
F002982	Coconut, cream, regular fat
F002991	Coconut, milk, canned, regular fat
F003998	Flour, rice
F006136	Oats, hulled, uncooked
F007598	Quinoa, uncooked
F008209	Seed, chia, dried
F005638	Milk, cow, fluid, skim (0.15% fat), added milk solids
F005637	Milk, cow, fluid, skim (0.15% fat)
F005598	Milk, cow, fluid, lactose-free, reduced-fat (1% fat)
F005614	Milk, cow, fluid, reduced-fat (1% fat)
F005621	Milk, cow, fluid, reduced-fat (1.5% fat), added omega 3 polyunsaturates
F005599	Milk, cow, fluid, lactose-free, regular fat (3.5% fat)
F005634	Milk, cow, fluid, regular fat (3.5% fat)
12012 ^1^	Seeds, hemp seed, hulled

^1^ Values for hemp were derived from the USDA Food database due to lack of availability.
